# Time-Limited Eating in Pediatric Patients with Obesity: A Case Series

**DOI:** 10.26502/jfsnr.2642-11000022

**Published:** 2019-09-20

**Authors:** Alaina P Vidmar, Michael I Goran, Jennifer K Raymond

**Affiliations:** Diabetes and Obesity Program, Center for Endocrinology, Diabetes and Metabolism, Department of Pediatrics, Children’s Hospital Los Angeles and Keck School of Medicine of USC, Los Angeles, CA, USA

**Keywords:** Obesity, Pediatrics, Time Limited Eating

## Abstract

**Background:**

Time Limited Eating (TLE) is an effective strategy for management of obesity in adults, but there is a paucity of data that have examined its use in the clinical management of children with obesity. A TLE approach involves interspersing normal daily caloric intake with periods of prolonged calorie restriction several times per week. TLE may actually be more feasible, non-stigmatizing, flexible and effective in children, especially for adolescents, compared to alternatives like daily caloric or macronutrient restriction. This is because TLE removes the need for intensive counting of daily calorie intake or macronutrient content and focuses on a straightforward task of consuming food during a pre-specified time period. Also it avoids periods of extended caloric restriction which may interfere with growth and/or risk evoking development of eating behaviors. This case series describes four patients who trialed a TLE approach in a clinical weight management clinic and describes BMI reduction at 4 months.

**Case Presentation:**

To date, 4 patients, ages 5–15, with varying underlying pathologies (i.e. Bardet Biedl Syndrome (BBS), previously healthy, craniopharyngioma and epilepsy) have tried a TLE type approach (16-hour fast/8-hour feed for 3–5 days per week) for 4 months and have demonstrated an average decrease in their BMI z-score compared to baseline of −0.24 SD. Patients and their families reported high degrees of satisfaction with this dietary approach.

**Conclusions:**

Families were very satisfied with the TLE intervention and reported it was feasible, flexible and sustainable to implement in a real life setting and associated with decreased zBMI. Further investigation is required to determine if this approach is effective in both the short and long term as a weight management technique.

## 1. Introduction

As the prevalence of pediatric obesity continues to rise, there remains a great need to identify effective, sustainable and feasible dietary interventions for this high risk population [1, 2]. The current clinical practice guidelines for the management of pediatric obesity recommend multi-disciplinary lifestyle intervention which combines behavior change, dietary interventions and physical activity [3, 4]. The dietary interventions historically have incorporated caloric restriction, macronutrient monitoring (carbohydrate control) and various other techniques in an attempt to reduce body mass and improve cardio metabolic outcomes; however most weight loss outcomes are modest and often difficult to maintain [3–5]. The success of any of these dietary strategies rests in the individual’s adherence to these recommendations in their real life settings and hinges on the patient’s developmental stage, family environment and personal preferences [6–8]. Therefore, further investigation into novel dietary approaches is warranted to increase the range of treatment options available to this population. Time limited eating (TLE) is a dietary intervention that has been reported to result in weight loss, decreased body fat and improvement in β-cell function [9, 10]. Novel dietary approaches like TLE have been shown to be effective for weight loss and improve glycemic control in adults with obesity. Interestingly, to date only one study has examined the impact of this intervention on weight outcomes in pediatric patients [10]. There exists various forms of TLE implementation [11–14].

All TLE approaches include periods of complete fasting that alternate with either ad libitum feeding or prescribed reduced energy intake. Literature in adults has revealed that this technique is as effective in reducing body mass and achieving weight loss as daily, continuous energy restriction [6, 15, 16]. Additionally, given the concrete, specific recommendation for time focused eating patterns; this approach may result in improved adherence in the pediatric population given that it does not require laborious tracking or skilled numeracy [10]. A recent study, the first reported to date, by Jebeile et al. evaluated the use of TLE on BMI change in adolescents and found it be a feasible, effective and acceptable dietary strategy in this population [10].

This case series explores the use of TLE as a clinical alternative dietary intervention for the management of pediatric obesity from one multi-disciplinary weight management clinic based in a Division of Pediatric Endocrinology at Children’s Hospital of Los Angeles. To date, 4 patients, ages 5–15 years, with varying underlying pathologies (i.e. Bardet Biedl Syndrome (BBS), previously healthy, craniopharyngioma and epilepsy) have tried a TLE type approach and have demonstrated an average decrease in their BMI z-score (zBMI) compared to baseline of −0.24 standard deviation (SD). Overall, the patient and families were very satisfied with the intervention and reported it was feasible, flexible and sustainable to implement in a real life setting.

## 2. Case Presentations

All 4 patients were referred to and evaluated in the multi-disciplinary weight management clinic at Children’s Hospital of Los Angeles. The current prevalence of childhood obesity in Southern California is 5–40% given the population demographic, socioeconomic status and ethnic composition [17]. The clinic consists of a pediatric endocrinologist, nutritionist, physical therapist and social worker. Each provider completed a thorough assessment at visit one with each patient and family and an individualized plan was obtained. Patients and their families returned to clinic at monthly intervals. Fasting lab tests were obtained at baseline and 6 months for clinical monitoring for each patient (Table 1). Each patient and family selected to implement some form of TLE approach, in which for 3–5 days per week they fasted for 12–16 hours per day. The remaining days of the week they adhered to an age appropriate healthy eating plan determined with assistance from the RD in clinic. No calorie counting or macronutrient monitoring was performed during this period. Adherence was monitored by self-reported dietary history, obtained at monthly intervals by the clinical team. Outcomes were measured at monthly increments from baseline to 4 months including: height, weight, BMI, BMI z-score (zBMI), quality of life and diet acceptability.

### 2.1 Case 1

5-year-old Hispanic female with history of Bardet Biedl syndrome (BBS) presented with concern for early onset weight gain in the setting of hyperphagia. She was born full term, via normal spontaneous vaginal delivery. Prenatal ultrasound at 36 weeks gestational age revealed enlarged kidney and extra digit. Delivery was uncomplicated. At 8 months of age, she was noted to have horizontal nystagmus followed by delayed speech noted at 24 months of age. Subsequent evaluation and testing revealed a heterozygous mutation of c. 273 c>G of BBS10 gene with predicted pCys91Trp mutation consistent with diagnosis of BBS. Shortly after diagnosis, she was enrolled in therapy services including physical, occupational and speech therapy. She was referred to endocrinology for assistance with weight management at the age of 4 due to parental concerns of worsening hyperphagia type symptoms and rapid weight gain over the past 9 months. Prior to the initial evaluation, the family had not implemented any form of structured eating plan for the patient and she was consistently eating frequently from 7 AM to 9 PM.

Initial evaluation revealed that the child did exhibit hyperphagia behaviors with significant behavioral responses to be refused food. The Family elected to implement a TLE approach for the whole family and limit eating to between 8 AM-5 PM, 5 days per week. The remaining two days the parents allowed for eating until 7 PM, which was the child’s bedtime. At the one month follow up visit, the parents reported that the child took very well to the TLE approach. They reported that the structured time of eating provided a guideline for the whole family and ensured all caregivers were consistent.

**Figure 1:**
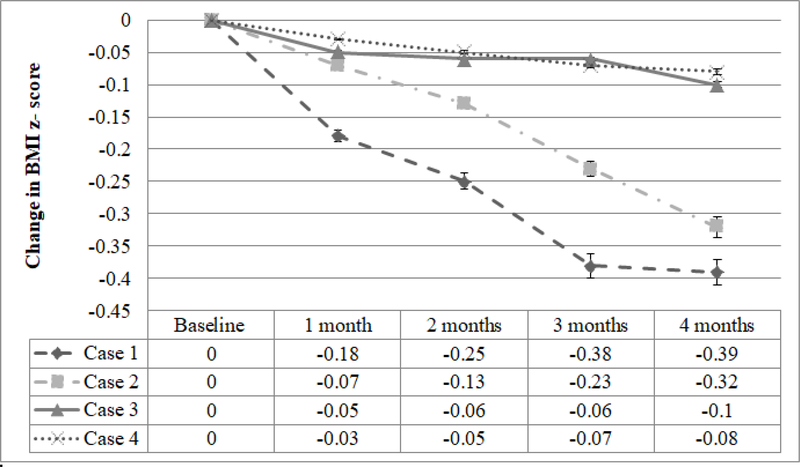
Change in BMI Z-score across intervention period compared to baseline across the 4 patients reported. All 4 patients exhibited a down trend in their zBMI at 4 months compared to baseline.

Additionally, the parents described that they were able to decrease the amount of food focused behavioral rewards they were administering for good behaviors during the fasting periods, especially in the evenings. The parents, older brother (age 9) and patient all were implementing the TLE approach together in the home. At the one month follow up, her zBMI has trended down by −0.1 SD. The family returned to all scheduled follow up visits and the zBMI continued to decrease with a −0.39 SD reduction at 4 months compared to baseline (See Figure 1). Overall, the child adapted very well to the intervention and per parents did not demonstrate any distress of behavioral concerns in relationship to the eating periods. Parents reported high levels of satisfaction with this approach and have continued it past the 4 months follow up.

### 2.2 Case 2

A 12-year-old, previously healthy Hispanic female, presented for excess weight gain over the past 12 months. The patient and father reported that she was a sedentary teen at baseline and had a history of eating frequently throughout the day and into the night due to emotional distress or boredom. The family had not attempted any lifestyle interventions to date as the parent-teen relationship was quite strained and it was difficult for either parent to encourage or promote appropriate, healthy eating habits. After evaluation from the team, it was identified that one of the biggest sources of discord between the patient and parents was that the teen did not want to eat breakfast. It was determined that a TLE approach with the eating window starting at 11 AM would support the patient’s desire to not consume breakfast and create a structure to her eating pattern 3 days per week.

The entire family (mother, father, 15 and 7 year old sister) decided to implement a TLE approach consisting of eating between 11 AM-7PM, 3 days per week and adhering to an age-appropriate healthy diet the remaining 4 days of the week. The family returned for the 1 month visit and the patient’s zBMI had trended down by −0.2 SD. She reported great satisfaction with the intervention and shared that by removing the battle over her breakfast consumption with her parents the parent-teen relationship was improving. Her zBMI decreased by 0.32 SD over the 4 months follow up period compared to baseline (Figure 1). She reported that the TLE approach was feasible and easy to adhere to and she enjoyed how her focus was not on the amount of food she was eating but the time of day she was eating. Per report, by removing the need for calorie counting or restriction of macronutrient content, she did not feel deprived and explained that it felt more like a lifestyle change than a traditional diet. The entire family, at the 4 month visit, reinforced this idea to the provider team.

### 2.3 Case 3

11-year-old Hispanic female with history of craniopharyngioma status post resection with subsequent multiple pituitary hormone deficiencies on replacement therapies including growth hormone, levothyroxine, hydrocortisone (at physiologic dose of 7 mg/m^2^/day) and desmopressin who presented with concern for excess appetite and weight gain for past 2 years with rapid weight gain for weight management treatment. She was diagnosed with non-alcoholic fatty liver disease and type 2 diabetes, controlled with stable dose Metformin (1000 mg BID) 12 months prior to presentation.

At initial visit, parents reported they had attempted caloric restriction, carbohydrate reduction and low fat dietary regimens without any change in her weight gain trajectory. The patient reported she had high levels of hunger and never experienced satiety. She reported she was consistently eating from 7 AM-11 PM most days due to her frequent hunger. Family was requesting alternative dietary intervention that did not require caloric monitoring or macronutrient recording. The patient and her parents elected to implement a TLE approach, limiting eating from 9 AM to 5 PM, 4 days per week and adhering to an age-appropriate healthy diet the remaining 4 days of the week without a specific caloric restriction.

At the 1 month visit, the patient reported the intervention was easy to implement and she didn’t feel she had to restrict herself during her eating window. Additionally, she reported that the structured eating time-period prevented her from overeating and she subjectively commented that she was experiencing more satiety than she had during her previous dietary intervention trials. Her zBMI decreased by 0.1 SD over a 4 month period (See Figure 1).

### 2.4 Case 4

15-year-old Hispanic female with neonatal cerebral hemorrhage, epilepsy, developmental delay and inability to ambulate presented for assistance with excessive weight gain in the setting of non-ambulation for the past 16 months. On presentation, entire family attended the visit. The patient had a great support system. The main barrier reported by the mother and father was that physical activity had always been the method utilized to control weight in their family and the patient’s inability to ambulate had resulted in rapid weight gain.

The entire family has trialed a dietary intervention focusing on raw foods, low fats and low carbohydrate intake for the previous 6 months with no BMI reduction noted. Dietary recall revealed that the patient was eating 16–17 hours per day, per report, secondary to her inability to ambulate. All six members of the family elected to implement a TLE approach in which the family only ate between 11 AM-7 PM, 5 days per week adhering to an age-appropriate healthy diet the remaining 2 days of the week without a specific caloric restriction.

At the 4 month visit, all family members reported weight loss and satisfaction with the intervention. The patient reported that restricting her eating period had enabled her to take on several new hobbies and focus on non-food based distractions and entertainment techniques. The patient reported that compared to her previous experience with caloric restriction, she found the TLE approach to be much simpler to implement and appreciated how it provided her with more autonomy as she could independently adhere to it despite her cognitive barriers. Her zBMI decreased by 0.16 SD over 4 months.

## 3. Discussion

This case series demonstrates that a TLE based approach has the potential to be a feasible, sustainable and effective dietary intervention in clinical management of pediatric obesity. The change in zBMI from baseline to 4 months in these four cases mirror that reported by a randomized control trial evaluating the feasibility, effectiveness, and acceptability of intermittent energy restriction (IER) in adolescents with obesity performed by Jubeile et al. which reported a decrease of −0.12 SD after a 6 month intervention period [10]. In comparison, the current expected BMI reduction in adolescents after various medical interventions are as follows: 1) participation in a 6 month multi-disciplinary clinical weight management interventions, −0.1 to −0.3 SD, 2) 6 month trial of an anti-obesity pharmacotherapy such as Topiramate, metformin or phentermine, −0.2 to −0.5 SD and 3) weight loss surgery, −0.5 to −1 SD. These results highlight that a TLE approach can result in BMI reduction over the short term and support further work exploring the use of this type of intervention for long term weight maintenance in the pediatric population. Additionally, these findings support data from the adult literature which has demonstrated that TLE results in weight loss and reduced adiposity after short term intervention periods.

The four patients and their families in this case series found the TLE approach easy to implement in the real life setting across a variety of ages, developmental stages and cognitive abilities. Parents reported that the approach provided flexibility that promoted adherence for all members of the family and accommodated busy schedules and unpredictable eating patterns. Further investigation is required to determine if a TLE approach may result in sustained weight control over time. Additionally, the use of prescriptive dietary interventions in the pediatric populations remains a controversial topic and requires well designed trials to explore how these types of interventions can be utilized to promote sustained habit change in this high risk population. All four cases reported presented with highly motivated families and patients which certainly played a role in the success of the intervention. The families were not only highly motivated and supportive of the patients’ treatment plan, they also participated themselves in the intervention. Previous reports have demonstrated that family participation in dietary intervention is a key component to BMI reduction in the children and adolescents [2]. The high level of satisfaction across the diverse patient profiles warrants further exploration to determine which specific pediatric obesity phenotype is best targeted with this intervention.

## 4. Conclusions

Overall, families were very satisfied with the TLE intervention and reported it was feasible, flexible and sustainable to implement in a real life setting and associated with decreased zBMI and therefore further investigation is required to determine if this approach is effective in both the short and long term and identify which patients this approach is best suited for as a weight management technique.

## Figures and Tables

**Table 1:** Anthropometry and biochemistry at baseline and 4 months in pediatric patients with obesity following a time limited eating dietary approach.

Lab Tests	Case 1: 5 yo		Case 2: 12 yo		Case 3: 11 yo		Case 4: 15 yo	
	Baseline	6 mo	Baseline	6 mo	Baseline	6 mo	Baseline	6 mo
Anthropometry								
BMI z score (SD)	2.57	2.18	2.47	2.15	2.64	2.54	2.24	2.16
BMI % 95^th^ percentile (percent)	125	110	140	120	150	140	110	95
Blood Pressure								
Systolic blood pressure, mm Hg	104	94	117	107	128	114	126	125
Diastolic blood pressure, mm Hg	54	55	58	53	85	76	76	73
Biochemistry (Plasma)								
Fasting Plasma Glucose, mg/dL	95	85	85	76	294	118	80	84
ALT, U/L	32	6	13	12	209	204	53	30
AST, U/L	32	46	14	15	158	209	34	33
Total Cholesterol, mg/dL	-	-	128	101	294	258	174	180
Triglyceride, mg/dL	-	-	141	90	224	188	200	153
HDL, mg/dL	-	-	42	45	30	33	26	26
LDL, mg/dL	-	-	58	51	167	110	120	125

## Abbreviations

BMI: Body mass index
zBMI: Body mass index Z score
%BMIp95: Percent over the 95^th^ percentile
ALT: Alanine aminotransferase

## References

[1] Hales CM, Fryar CD, Carroll MD, Trends in Obesity and Severe Obesity Prevalence in US Youth and Adults by Sex and Age, 2007–2008 to 2015–2016. Jama 319 (2018): 1723–1725. 29570750 10.1001/jama.2018.3060 PMC5876828

[2] Tu AW, Watts AW, Chanoine JP, Does parental and adolescent participation in an e-health lifestyle modification intervention improves weight outcomes? BMC Public Health 17 (2017): 352. 28438202 10.1186/s12889-017-4220-0 PMC5402679

[3] Styne DM, Arslanian SA, Connor EL, Pediatric Obesity-Assessment, Treatment, and Prevention: An Endocrine Society Clinical Practice Guideline. J Clin Endocrinol Metab 102 (2017): 709–757. 28359099 10.1210/jc.2016-2573 PMC6283429

[4] Barlow SE. Expert committee recommendations regarding the prevention, assessment, and treatment of child and adolescent overweight and obesity: summary report. Pediatrics 120 (2007): 164–192. 10.1542/peds.2007-2329C 18055651

[5] August GP, Caprio S, Fennoy I, Prevention and treatment of pediatric obesity: an endocrine society clinical practice guideline based on expert opinion. J Clin Endocrinol Metab 93 (2008): 4576–4599. 18782869 10.1210/jc.2007-2458 PMC6048599

[6] Kroeger CM, Trepanowski JF, Klempel MC, Eating behavior traits of successful weight losers during 12 months of alternate-day fasting: An exploratory analysis of a randomized controlled trial. Nutr Health 24 (2018): 5–10. 29353535 10.1177/0260106017753487 PMC7183822

[7] Andela S, Burrows TL, Baur LA, Efficacy of very low-energy diet programs for weight loss: A systematic review with meta-analysis of intervention studies in children and adolescents with obesity. Obes Rev 20 (2019): 871–882. 30734459 10.1111/obr.12830

[8] Gow ML, Ho M, Burrows TL, Impact of dietary macronutrient distribution on BMI and cardiometabolic outcomes in overweight and obese children and adolescents: a systematic review. Nutr Rev 72 (2014): 453–470. 24920422 10.1111/nure.12111

[9] Ganesan K, Habboush Y, Sultan S. Intermittent Fasting: The Choice for a Healthier Lifestyle. Cureus 10 (2018): 2947. 10.7759/cureus.2947 PMC6128599 30202677

[10] Jebeile H, Gow ML, Lister NB, Intermittent Energy Restriction Is a Feasible, Effective, and Acceptable Intervention to Treat Adolescents with Obesity. J Nutr 149 (2019): 1189–1197. 31006807 10.1093/jn/nxz049

[11] Fitzgerald KC, Vizthum D, Henry-Barron B, Effect of intermittent vs. daily calorie restriction on changes in weight and patient-reported outcomes in people with multiple sclerosis. Mult Scler Relat Disord 23 (2018): 33–39. 29753994 10.1016/j.msard.2018.05.002 PMC6107078

[12] Gabel K, Hoddy KK, Haggerty N, Effects of 8-hour time restricted feeding on body weight and metabolic disease risk factors in obese adults: A pilot study. Nutr Healthy Aging 4 (2018): 345–353. 29951594 10.3233/NHA-170036 PMC6004924

[13] Mattson MP, Longo VD, Harvie M. Impact of intermittent fasting on health and disease processes. Ageing Res Rev 39 (2017): 46–58. 27810402 10.1016/j.arr.2016.10.005 PMC5411330

[14] Jane L, Atkinson G, Jaime V, Intermittent fasting interventions for the treatment of overweight and obesity in adults aged 18 years and over: a systematic review protocol. JBI Database System Rev Implement Rep 13 (2015): 60–68. 10.11124/jbisrir-2015-2363 26571283

[15] Gabel K, Hoddy K, Burgess HJ, Effect of 8-hour time-restricted feeding on sleep quality and duration in adults with obesity. Appl Physiol Nutr Metab 44 (2019): 903–906. 30802152 10.1139/apnm-2019-0032

[16] Anton SD, Moehl K, Donahoo WT, Flipping the Metabolic Switch: Understanding and Applying the Health Benefits of Fasting. Obesity (Silver Spring) 26 (2018): 254–268. 29086496 10.1002/oby.22065 PMC5783752

[17] Shih M, Dumke KA, Goran MI, The association between community-level economic hardship and childhood obesity prevalence in Los Angeles. Pediatr Obes 8 (2013): 411–417. 23239616 10.1111/j.2047-6310.2012.00123.x

